# Evaluation of Interfacial Structure and Interaction between Alkylamine-Modified Ag and Organic Solvent via Molecular Dynamics Simulations

**DOI:** 10.1021/acs.langmuir.5c06493

**Published:** 2026-05-12

**Authors:** Hiroto Sawauchi, Yuto Sato, Takamasa Saito, Eita Shoji, Shohei Yamashita, Yohei Okada, Atsuki Komiya, Masaki Kubo

**Affiliations:** † Department of Chemical Engineering, Graduate School of Engineering, 13101 Tohoku University, 6-6-07 Aramaki, Aoba-ku, Sendai, Miyagi 980-8579, Japan; ‡ Department of Mechanical Systems Engineering, Graduate School of Engineering, Tohoku University, 6-6-01 Aramaki, Aoba-ku, Sendai, Miyagi 980-8579, Japan; § Department of Applied Biological Science, Tokyo University of Agriculture and Technology, 3-5-8 Saiwai-cho, Fuchu, Tokyo 183-8509, Japan; ∥ Institute of Fluid Science, Tohoku University, 2-1-1 Katahira, Aoba-ku, Sendai, Miyagi 980-8577, Japan

## Abstract

Controlling the dispersion stability of surface-modified nanoparticles in suspensions is essential for their successful industrial application. However, the structures and interactions at the interface between the surface-modified nanoparticles and organic solvents remain poorly understood. In this study, the effects of the surface coverage and solvent species on the interfacial structure and interactions between the dodecylamine-modified Ag slab and organic solvents were investigated using all-atom molecular dynamics simulations. Toluene and methanol were selected as typical nonpolar and polar solvents, respectively. At higher surface coverages, the ligand exhibited more ordered and oriented structures. In toluene (a good solvent for dodecylamine-modified Ag), more ligands extended toward the solvent phase and more solvent molecules penetrated the ligand layer compared to methanol (a poor solvent for dodecylamine-modified Ag). As a result, the interaction between the solvent and the alkyl chain of the ligand for toluene was stronger than that for methanol. The degree of penetration and the interaction in both solvent systems varied with the surface coverage, showing a maximum in penetration and a minimum in interaction energy at 50% coverage. These findings provide insights into the design of ligands to improve the dispersion stability of surface-modified nanoparticles in organic solvents.

## Graphical abstract

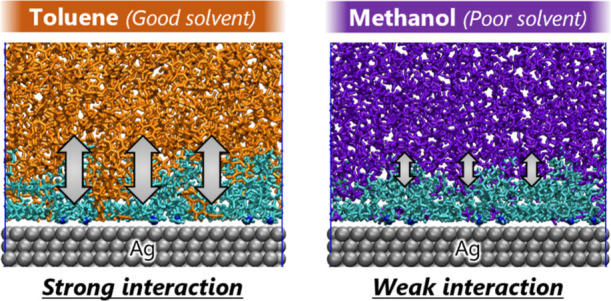

## Introduction

Silver (Ag) nanoparticles exhibit physical and chemical properties that differ significantly from those of bulk materials and have excellent optical,^1^ electrical,^2^ catalytic,^3^ and antibacterial^4^ properties. These features provide attractive applications of Ag nanoparticles in various fields such as electronics, catalysis, energy, and medicine. However, owing to their high surface energy, nanoparticles are prone to aggregation, which reduces their performance. Additionally, the properties of nanoparticles are influenced by not only their core size and shape but also their dispersion states. Therefore, controlling the dispersion state of nanoparticles is essential for enhancing their material performance and enabling practical applications.

Surface modification using organic ligands is a common technique to control the dispersion state in solvents.^5–7^ Thiol- and amine-type ligands are often used for metal nanoparticles (e.g., Au and Ag). It is widely known that nanoparticles modified with aliphatic ligands can be dispersed in hydrophobic (nonpolar) solvents, so-called “like dissolves like”. For example, it has been reported that dodecylamine-modified Ag nanoparticles dispersed in toluene, while aggregated in methanol.^8^ Moreover, the dispersion state of nanoparticles has been reported to be improved using ligands of amphiphilic (e.g., a combination of ethylene glycol chains and alkyl chains),^8–10^ kinked (e.g., *cis* double bonds),^11^ and branched structures.^12,13^ Therefore, the design of appropriate ligands considering the affinity and molecular structure is important for the development and utilization of highly dispersed nanoparticles.

The dispersion and aggregation mechanisms of the surface-modified nanoparticles are still under debate. Recent experimental and computational studies have proposed that the bundling of ligands promotes the aggregation of nanoparticles in solvents.^14–17^ Additionally, the dispersion state of the nanoparticles is influenced not only by the miscibility of the ligand and solvent species but also by the molecular structure and size of the solvent.^17–20^ These behaviors cannot be explained by conventional models of interactions between the surface-modified nanoparticles.^21^ However, it remains difficult to directly investigate the actual structure and interactions between the surface-modified nanoparticles and the solvent. Therefore, the interfacial structure and interactions are not necessarily understood.

Molecular dynamics (MD) simulations are an effective method for visualizing and evaluating nanoscale interfacial structures and interactions.^22–27^ In order to design appropriate ligands, it is important to obtain fundamental knowledge about interfacial structures and interactions in detail using MD simulations. In this study, all-atom MD simulations were performed to evaluate the interfacial structures and interactions between alkylamine-modified Ag and organic solvents. The effects of surface coverage and polarity of organic solvents on the interfacial structures and interactions were investigated. Note that the simulation results correspond to local flat interfaces, not including the effects of curvature, facets, edges, and so on.

## Simulation Method

### Simulation Model

To represent the atop-site adsorption of amine groups on the Ag surface,^28^ the AgP-CHARMM force field^29,30^ was used for the interactions between Ag and organic molecules. This force field employs the rigid-rod dipole approach and the virtual interaction sites on the Ag surface to capture the electric metal polarization. Image charges with a mass of 0.5u were arranged and connected to each Ag atom at a distance of 0.7 Å. The partial charge of Ag atoms and image charges were −0.308e and 0.308e, respectively. Virtual sites were arranged at the hollow sites on the Ag surface and interacted with organic molecules. Dodecylamine was used as the alkylamine ligand. Toluene and methanol were selected as typical nonpolar and polar solvents and were good and poor organic solvents, respectively, for the dodecylamine-modified Ag nanoparticles.^8^ Since this study focuses on the interactions between ligand and solvent, the all-atom optimized potentials for liquid simulation (OPLS-AA) force fields^31–33^ were applied to dodecylamine, toluene, and methanol, which is suitable for describing the physicochemical properties of small organic molecules. The L-OPLS-AA parameters^34^ were applied to the alkyl chain of dodecylamine to better represent the properties for long hydrocarbons. It was validated that the adsorption energies using a combination of AgP-CHARMM and (L-)­OPLS were approximately similar to those using a combination of AgP-CHARMM and CHARMM. The Lennard-Jones (LJ) interaction parameters between AgP (virtual sites and bulk Ag atoms) and amine and hydroxy groups followed the determined parameters in the AgP-CHARMM force field. The Lorentz–Berthelot combining rule was applied to other LJ interaction parameters between AgP and (L-)­OPLS-AA atoms. Following the OPLS-AA force field,^31^ the geometric rule was used for the LJ interaction parameters of (L-)­OPLS-AA atoms. Intramolecular nonbonded interactions for dodecylamine, toluene, and methanol were considered for atom pairs separated by three or more bonds. The scaling factor was set to 0.5 for 1,4-interactions.^31^

A planar interface model was used to investigate the interface between surface-modified nanoparticles and organic solvents. Figure 1a shows the simulation model of the dodecylamine-modified Ag/organic solvent system. Periodic boundary conditions were applied in the *x-* and *y*-directions. Following the previous study of the AgP-CHARMM force field,^29^ the Ag slab was constructed using a lattice constant of 4.165 Å with five layers thick. Focusing on the homogeneous spherical nanoparticles, the most common (111) facet was selected as a representative surface. The surface area of the slab was 5.3 × 5.1 nm^2^ greater than the sum of the ligand chain length and the two times of the cutoff distance for short-range interactions. An organic solvent film was arranged above the dodecylamine-modified Ag slab. A piston wall was placed above the organic solvent film to control the pressure perpendicular to the interface and prevent solvent evaporation.

**Figure 1:**
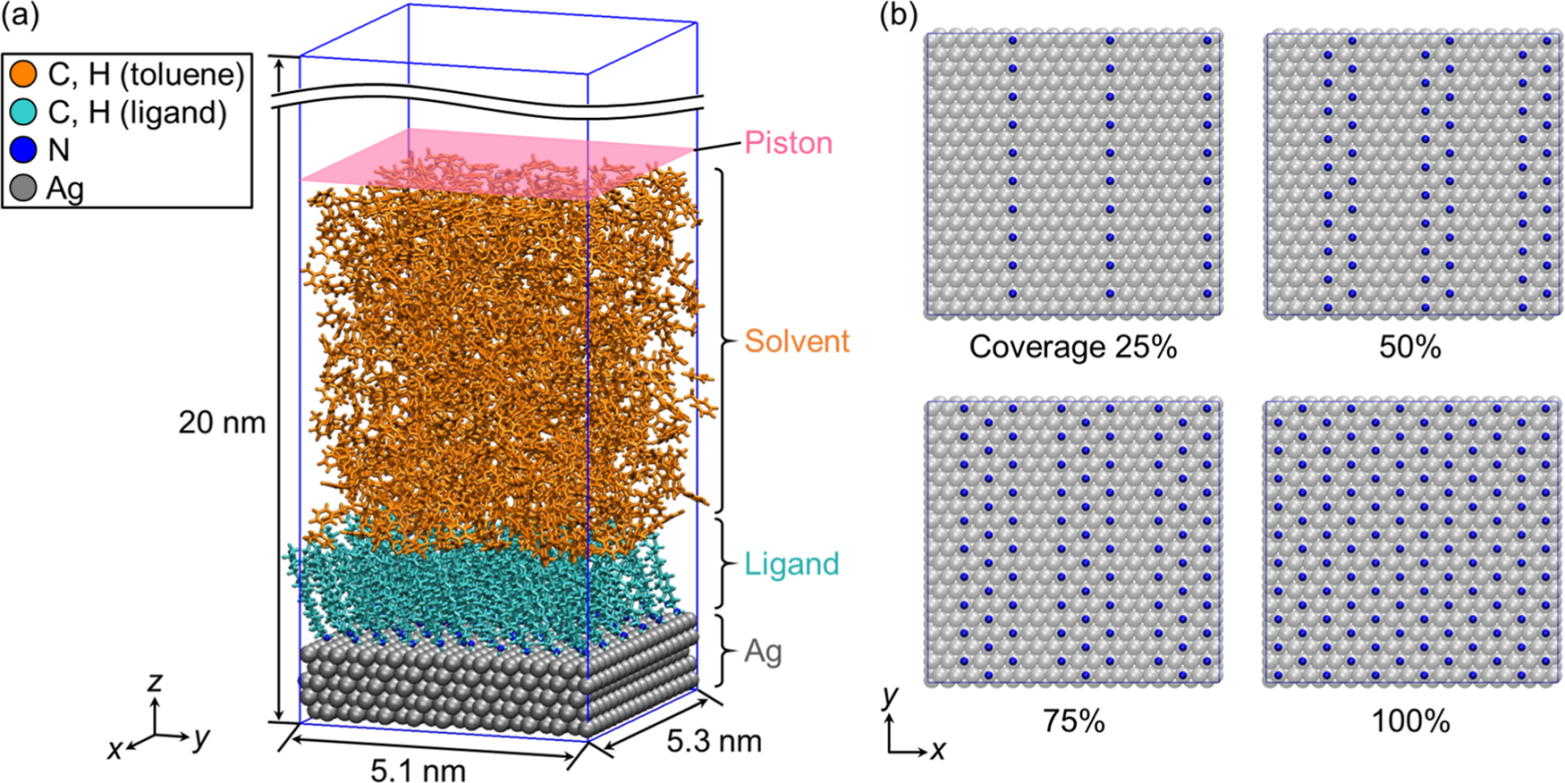
(a) Simulation model of the dodecylamine-modified Ag/organic solvent system. The surface coverage is 100% and the solvent is toluene, as an example. Only Ag atoms are displayed for clarity. (b) The surface modification patterns of dodecylamine on the Ag(111) surface for various surface coverages. Ag atoms are drawn transparently for clarity.

Figure 1b shows the surface modification patterns of dodecylamine on the Ag(111) surface for various surface coverages. The nitrogen atom of the ligand was arranged directly above the Ag atom, i.e., at the atop site. In the AgP-CHARMM model, the amine ligand adsorbs on the atop site of the Ag surface; however, the amine ligand on the Ag surface is able to diffuse laterally and rearrange without constraint. To systematically examine the effect of surface coverage on the interfacial structure, each nitrogen atom was tethered to the position on the atop sites using a spring force with an arbitrary spring constant (10 kcal/(mol·Å^2^)) along the *xy*-plane, limiting the degree of freedom such as lateral diffusion and rearrangement of the ligand on the Ag surface. This constraint may not represent the actual adsorption behavior of the ligand but can clearly capture the ligand behavior for nonpolar and polar solvents with different surface coverages. Since there are no references regarding adsorption structures of alkylamines on the Ag(111) surface, each ligand was assumed to be arranged in a hexagonal $\left(\sqrt{3}\times \sqrt{3}\right)R30^{{}^{\circ}}$ pattern.^35^ In this pattern, there was a coverage density of 4.44 molecules/nm,^2^ defined as a surface coverage of 100%. In the case of 25–75% surface coverage, the ligands were arranged in stripe patterns. The number of single rows consisting of 10 ligands along the *y*-direction varied with an increase in the surface coverage. This configuration was adapted from a previous report that dodecylamine molecules were adsorbed and arranged on the Au(111) surface as ordered domains of the stripe phase.^36^ Note that such a model was very limited because the assumption of transferring adsorption structures from Au to Ag is not justified by experimental work or density functional theory calculation. Nevertheless, this approach provides a consistent setting to investigate the effect of surface coverage of the ligand on the interfacial structure and interaction.

### Simulation Protocol

Initially, *NVE* ensemble simulation of the AgP slab was performed for 60 ps. Then, dodecylamine ligands were arranged on the Ag surface, and *NVT* ensemble simulations of the dodecylamine-modified Ag were performed at 298.15 K for 1 ns. Next, 914 toluene molecules or 2397 methanol molecules were packed into a 5.3 × 5.1 × 6.0 nm^3^ region using PACKMOL.^37^ These quantities were defined based on experimental densities of 0.862 g/cm^3^ (toluene) and 0.786 g/cm^3^ (methanol).^38^ *NVT* ensemble simulations of the organic solvent films were performed at 298.15 K for 3 ns as a vapor/liquid coexisting system under all periodic boundary conditions. Finally, the dodecylamine-modified Ag and solvent film were combined, and the equilibration of the interfacial system was performed at 298.15 K. The total equilibration time depended on the surface coverage: 50 ns for 25% and 75% surface coverage, 60 ns for 50% surface coverage, and 10 ns for 100% surface coverage. The equilibrium of the system was confirmed from the convergence of potential energies and the visualization of interfacial structure. The simulations were performed for three different initial configurations.

A detailed description for computational techniques is provided here. The temperature of ligand and solvent was maintained using a Nosé–Hoover style thermostat^39^ with a damping coefficient of 100 fs. The temperature of the AgP slab was not controlled. The pair of Ag atoms and image charges was treated as rigid bodies that were allowed to rotate but not translate. The Ag atoms were virtually fixed in the system, as the ratio of mass of Ag to the virtual charge was approximately 216:1. The pressure perpendicular to the interface was maintained at 1 atm using a piston wall above the solvent film. The detail of the piston wall is described in the Supporting Information. Initial velocities were given to atoms of the ligand and solvent with a Gaussian distribution at desired temperature. The reversible reference system propagator algorithm (r-RESPA) method^40^ was applied to numerically solve the equations of motion for atoms. A time step was set to 1 and 0.5 fs for the inter- and intramolecular interactions, respectively. The cutoff distance for the LJ and real-space Coulombic interactions was set to 15 Å. The particle–particle–particle–mesh (PPPM) algorithm^41^ was used to calculate the long-range Coulombic interactions. A modified scheme in the three-dimensional PPPM summation technique was applied to calculate the long-range Coulombic interactions for systems with a slab geometry that were periodic in *x*- and *y*-directions but nonperiodic in *z*-direction.^42^ This method treats the *z*-direction as periodic and effectively turns off slab–slab interactions by inserting empty volume between simulation boxes and removing dipole–dipole interactions between periodic images. The Large-scale Atomic/Molecular Massively Parallel Simulator (LAMMPS)^43^ was used to perform all MD simulations.

### Analysis

Simulation results were visualized using visual molecular dynamics (VMD) software.^44^ The tilt angle of the dodecylamine ligands with respect to the surface normal was calculated to examine the orientation and ordering of the ligand molecules. The chain axis of the ligand was defined as the vector joining the nitrogen and terminal carbon atom. A smaller tilt angle indicates a more-uptight chain.

To examine the interfacial structure, the mass density distribution was analyzed along the *z*-direction normal to the interface. The simulation box was evenly sliced into the spatial bin of 0.1 Å thickness along the *z*-direction, and the density distribution was obtained by computing the mass density for each bin, i.e., total mass of target atoms in bin per bin volume. The bin size is comparable to some previous studies.^45–49^

To quantify the overlap between the ligand layer and the solvent phase, that is, the degree of solvent penetration into the ligand layer, the overlap parameter, *P*_o_, was calculated as follows:^24^

1 $$P_{o}=\int_{0}^{\infty }\rho_{ligand}\left(z\right)\rho_{solvent}\left(z\right)dz$$

where ρ_ligand_ and ρ_solvent_ are mass densities of ligands and solvents, respectively. Some ways of quantifying the overlap have been discussed in previous studies.^50,51^ eq 1 was used to evaluate the overlap simply and directly without any normalization. Therefore, this overlap parameter has a unit, but the physical meaning is ignored in this study. A larger *P*_0_ indicates a greater degree of overlap, i.e., a higher degree of solvent penetration into the ligand layer.

To evaluate the interfacial affinity, the interaction energy was calculated as the sum of LJ and Coulombic potential energies between the ligand and solvent atoms. More negative values indicate stronger attractive interactions, i.e., better affinity. The rigorous approach to evaluate the interfacial affinity is the free energy calculation such as previous studies using thermodynamic integration methods.^22,52–54^ If the free energy is calculated, the contribution of interaction energy and entropy can be discussed.^26,55^ However, the accurate calculation for such soft interfaces still takes much computational cost owing to the need of many data points fully converged under the quasistatic process.^22^ Therefore, the calculation of the interaction potential energy was selected as the simple and practical approach.

The analysis was conducted using the last 5 ns data of equilibration simulation. The obtained data was divided into five blocks to calculate the block mean for each block. The overall average and standard deviation were calculated using all block means including the sampling data of different initial configurations, i.e., 15 block means for each surface coverage. Comparisons between calculated values was evaluated by 95% confidence intervals (95%CI)^56^ and *p*-values using Welch’s *t*-test. The details of these analyses are shown in the Supporting Information.

## Results and Discussion

### Tilt Angles of the Ligand

Figure 2 shows representative snapshots in the vicinity of the equilibrated interface between dodecylamine-modified Ag and solvents for various surface coverages. For both toluene and methanol systems, the higher the surface coverage was, the more upright the ligand was. A penetration of solvent into the ligand layer appeared at surface coverage of 25–75%. The difference in the interfacial structure is described later in detail.

**Figure 2:**
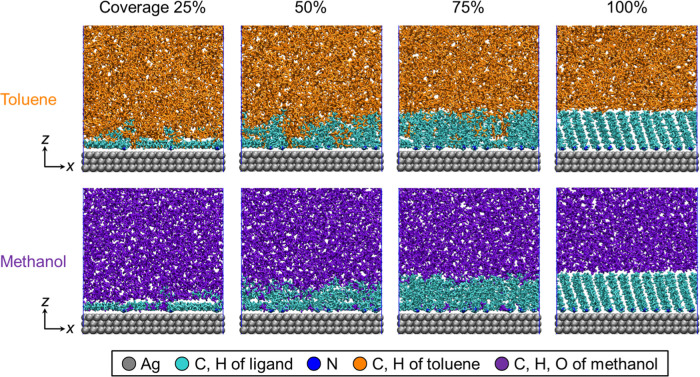
Representative snapshots in the vicinity of the equilibrated interface between dodecylamine-modified Ag and solvents for various surface coverages.

Figure 3 shows average tilt angle distributions of dodecylamine ligands for various surface coverages. At a surface coverage of 25%, a higher peak appeared at approximately 80–85° in both solvent systems. This result indicates that most of the ligands lay on the Ag surface. A smaller peak also appeared at approximately 40–60°. This is because some ligands extended toward the solvent phase, as shown in Figure 2. At a surface coverage of 50%, a broad peak appeared at approximately 20–60°, and the peak at approximately 80–85° became smaller than that for 25% surface coverage. This indicates that more ligands stood up and fewer ligands lay on the Ag surface. At a surface coverage of 75%, the peak mainly appeared at approximately 15–20°, but the peak barely appeared at approximately 80–85°. This is because most of the ligands stood up toward the solvent phase. At a surface coverage of 100%, the distribution was sharp and unimodal. This indicates a densely packing and high ordering of the ligands. The average tilt angle for 100% surface coverage was approximately 31°. This value is comparable to the tilt angle of alkylamine self-assembled monolayers on the Au(111) surface (approximately 30°).^57^ At all surface coverages, the tilt angle distribution of the ligand in the toluene system was lower than that in the methanol system. This indicates the alkyl chain of the ligand had a higher affinity to toluene than methanol, orienting toward the toluene phase. The extension and shrinkage of the ligand were confirmed by calculating the end-to-end distance of the ligand (see Figure S1 in the Supporting Information).

**Figure 3:**
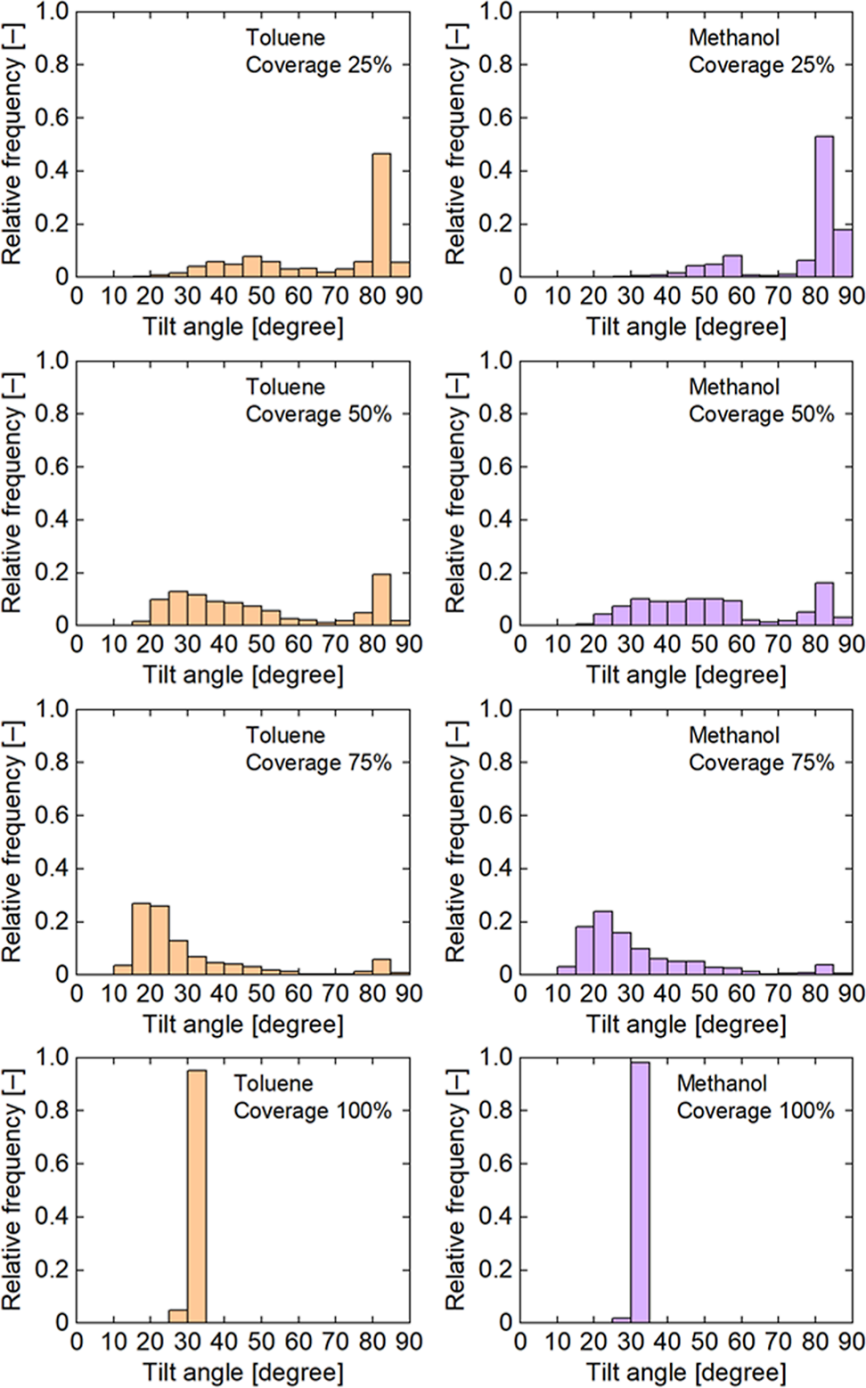
Average tilt angle distributions of dodecylamine ligands for various surface coverages.

### Density Distributions and Overlap Parameter

Figure 4 shows density distributions of the ligand and solvents near the interface as a function of the distance from the Ag surface for various surface coverages. The density of the solvent converged to its bulk value in the region far away from the interface. At the surface coverage of 25–75%, the distributions of the ligand and the solvent overlapped near the interface, proving the solvent penetration into the ligand layer as shown in Figure 2. At the surface coverage of 25%, the peaks of the solvent distributions below and above *z* = 0.55 nm indicate the solvent molecules adsorbing on the Ag surface and structuring on the ligand layer, respectively. At the surface coverages of 50% and 75%, the overlap of the distributions for methanol appears to be smaller than that for toluene. At the surface coverage of 75%, however, the peak of the methanol below *z* = 0.55 nm was higher than that of the toluene. This is because methanol is a polar solvent and has low affinity for alkyl chain of the ligand but high affinity for the Ag surface, a metallic and highly polarizable substance. At the surface coverage of 100%, the overlap of the distributions did not appear owing to the densely packing of the ligand. In addition, the solvent distribution oscillated in the vicinity of the interface, indicating the structurization of the solvent molecules on the ligand layer. The degree of overlap between the ligand layer and the solvent is quantitatively discussed in the next paragraph.

**Figure 4:**
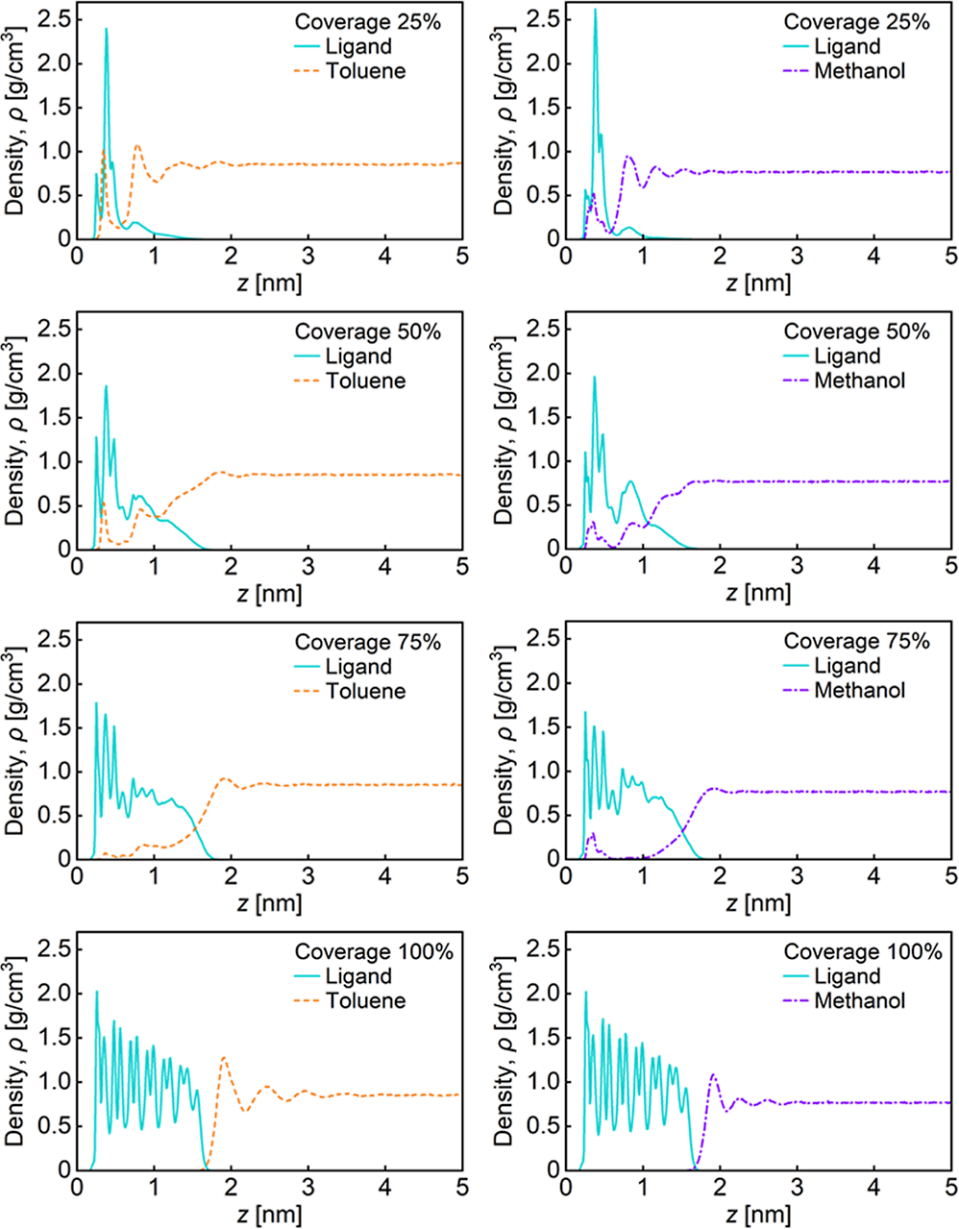
Density distributions of the ligand and solvents near the interface as a function of the distance from the Ag surface for various surface coverages.

Figure 5 shows the relationship between the surface coverage and overlap parameter for different solvent systems. Except for the surface coverage of 100%, the overlap parameters for toluene were higher than those for methanol, with a statistically significant difference (*p* < 0.05), showing the higher penetration of toluene than methanol. In both solvent systems, the overlap parameter was the largest at a surface coverage of 50%. This indicates that more ligands result in more contact area between the ligand and the solvent for low surface coverages and that more ligands result in less space to interact with the solvent for high surface coverages. Therefore, the difference in the degree of solvent penetration was confirmed by calculating the overlap parameter.

**Figure 5:**
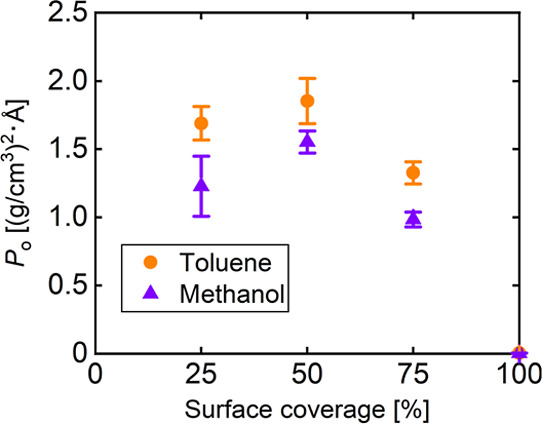
Relationship between the surface coverage and the overlap parameter for different solvent systems. Error bars represent the standard deviations.

### Interaction Energy

Figure 6 shows the effect of surface coverage on the interaction energy between the dodecylamine ligand and the solvent. In both solvent systems, the interaction energy was the most negative at a 50% surface coverage. This is because the degree of solvent penetration was the highest at 50% surface coverage, as shown in Figure 5. At the surface coverage of 100%, the interaction energy of toluene was more negative than that of methanol, with a statistically significant difference (*p* < 0.05), indicating that toluene has a higher affinity with the terminal methyl group of the ligand than methanol. However, the difference in interaction energy between toluene and methanol was not significant at the surface coverage of 50% (*p* > 0.05) and remained small at the surface coverage of 25 and 75%, despite being statistically significant (*p* < 0.05). This is because methanol molecules were adsorbed onto the Ag surface, and consequently, the calculated interaction energy of methanol included a strong interaction between methanol and the amine groups of the ligands. This is confirmed by the interaction energy between the Ag surface and solvent shown in Figure S2.

**Figure 6:**
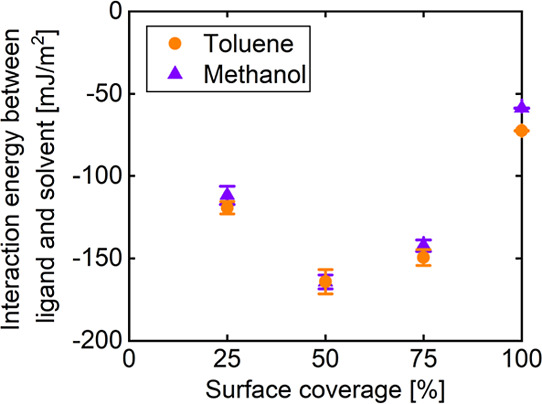
Effect of surface coverage on interaction energy between the dodecylamine ligand and solvent. Error bars represent the standard deviations.

Here, the effect of the headgroup of the ligands on the affinity between the surface-modified inorganic solid and the solvent is discussed. The dispersion state of dodecanethiol-modified gold nanoparticles in polymers was not predicted by the solubility parameter between dodecanethiol and the polymer but rather by that between dodecane and the polymer.^58^ Carboxylic acid-modified nanoparticles did not disperse in carboxylic acid but dispersed in similar alkanes.^18^ These reports suggest that the interaction with the tail group of the ligand, excluding the polar headgroup, is dominant for interaction between surface-modified nanoparticles and solvent. This is because the polar headgroup is directly bonded to the surface of the nanoparticles and the tail group is exposed to the solvent. On the basis of this idea, the interaction energy between the alkyl chain of the ligand and the solvent should be calculated, excluding the amine group of the ligand.

Figure 7 shows the effect of surface coverage on the interaction energy between the alkyl chain of the ligand and the solvent for the different solvent systems. For all surface coverages, the interaction energy of toluene was more negative than that of methanol, with a statistically significant difference (*p* < 0.05). The interaction energy was the most negative at the surface coverage of 50% in both solvent systems, as shown in Figure 6. This result is consistent with the trend of the overlap parameter in Figure 5, indicating that the interfacial affinity in toluene is higher than that in methanol. Therefore, the difference in the interfacial affinity for toluene and methanol was confirmed by evaluating the degree of solvent penetration and the interaction energy between the alkyl chain of the ligands and the solvent. The contributions of LJ and Coulombic terms are shown in Figure S3.

**Figure 7:**
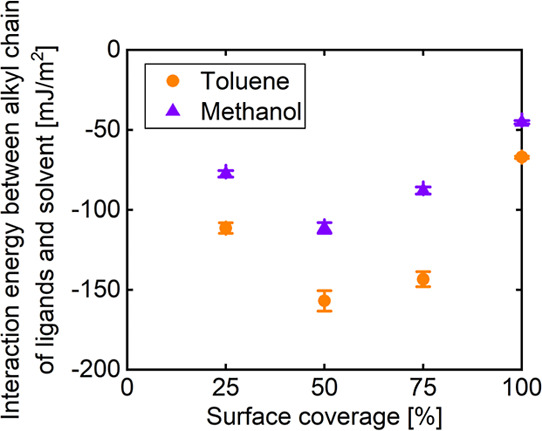
Effect of surface coverage on interaction energy between the alkyl chain of the ligand and the solvent for different solvent systems. Error bars represent the standard deviations.

## Discussion

The solvent penetration and interaction, that is, interfacial affinity, reached a maximum at around 50% surface coverage in both solvent systems. It is qualitatively understandable because excessive ligands reduce the accessible space for the solvents to penetrate the ligand layer. A nonlinear dependence of the interfacial affinity on the surface coverage was also reported by previous studies of MD simulations for interfaces between surface-modified solid and liquid (polymer). However, it is not obvious whether the optimal surface coverage is general to other systems. The optimal value is likely to depend on the solvent, ligand, and surface-modification pattern. For example, according to previous studies of MD simulations, decanoic acid-modified Al_2_O_3_ in *n*-hexane shows higher affinity and ordering structure at 75% surface coverage,^22^ whereas that in polypropylene shows higher penetration and affinity at 25% surface coverage.^24^ More investigations are necessary to understand the dominant driving factors of interfacial affinity depending on the molecular species and surface coverage in detail.

The results of the solvent penetration and interaction support the experimental result that dodecylamine-modified spherical Ag nanoparticles of 8 nm disperse in toluene and aggregate in methanol.^8^ Moreover, the ligand layer in toluene was more extended toward the solvent phase than that in methanol. It suggests that the thicker ligand layer also contributes to the steric hindrance between nanoparticles, i.e., the stable dispersion of nanoparticles. However, it is noted that the current simulations correspond to local flat interfaces on the ideal (111) surface, not including the effects of curvature, facets, and edges. A higher surface curvature of nanoparticles, i.e., smaller nanoparticle size, can lead to a greater degree of solvent penetration^59,60^ and more ordered structure of the ligand,^61^ which are sensitive to higher curvature. Ligands on facets are likely to be more disordered than those on the periodic flat slab.^62^ Furthermore, the current simulations were performed with the restrained configurations of ligands. Previous MD studies have reported that the amine ligands form the bilayer structure on Cu nanocrystals.^27^ Therefore, it remains a challenge to elucidate the relationship between the interfacial structure and the dispersion stability of surface-modified nanoparticles.

## Conclusions

All-atom molecular dynamics simulations were conducted to investigate the effects of surface coverage and solvent species on the interfacial structure and interactions between the dodecylamine-modified Ag slab and the organic solvents. Toluene and methanol were used as typical nonpolar and polar solvents, respectively.

The higher the surface coverage, the more upright and ordered the ligand. More ligands in toluene extended toward the solvent phase than those in methanol. In both solvents, the degree of the solvent penetration and the interaction energy between the alkyl chain of the ligand and the solvent were the highest and the most negative at 50% surface coverage, respectively. For all surface coverages, the degree of the solvent penetration and the interaction energy in toluene were higher and more negative than those in methanol, respectively. These results indicate that the interfacial affinity in toluene is greater than that in methanol.

The present simulations were performed for local flat interfaces under the constrained configurations of ligands. However, the simulation results support the previous experimental result of the dispersion stability of dodecylamine-modified Ag nanoparticles in the solvent.^8^ This study suggests that the interactions between surface-modified nanoparticles and organic solvents can be enhanced by selecting an appropriate amount of surface coverage and solvent species to control solvent penetration into the ligand layer.

## Supplementary Material



## Notes

Details of the piston wall setting, distributions of average end-to-end distance of dodecylamine ligands, interaction energy between Ag and solvent, and decomposition of interaction energy into LJ and Coulombic terms (PDF)

## References

[1] Kelly K. L., Coronado E., Zhao L. L., Schatz G. C. (2003). The Optical Properties
of Metal Nanoparticles: The Influence of Size, Shape, and Dielectric
Environment. J. Phys. Chem. B.

[2] Chen D., Qiao X., Qiu X., Chen J. (2009). Synthesis and electrical
properties of uniform silver nanoparticles for electronic applications. J. Mater. Sci..

[3] Dong Y., Gao Z.-W., Yang K.-F., Zhang W.-Q., Xu L.-W. (2015). Nanosilver
as a new generation of silver catalysts in organic transformations
for efficient synthesis of fine chemicals. Catal.:Sci.
Technol..

[4] Tang S., Zheng J. (2018). Antibacterial Activity
of Silver Nanoparticles: Structural Effects. Adv. Healthcare Mater..

[5] Heinz H., Pramanik C., Heinz O., Ding Y., Mishra R. K., Marchon D., Flatt R. J., Estrela-Lopis I., Llop J., Moya S., Ziolo R. F. (2017). Nanoparticle decoration
with surfactants: Molecular interactions, assembly, and applications. Surf. Sci. Rep..

[6] Calvin J. J., Brewer A. S., Alivisatos A. P. (2022). The role
of organic ligand shell
structures in colloidal nanocrystal synthesis. Nat. Synth..

[7] Iijima M., Kamiya H. (2009). Surface Modification
for Improving the Stability of
Nanoparticles in Liquid Media. KONA Powder Part.
J..

[8] Maeta N., Kamiya H., Okada Y. (2018). Direct Monitoring
of Molecular Events
at the Surface: One-Step Access to Flexibly Stable Colloidal Ag Nanoparticles. Langmuir.

[9] Okada Y., Ishikawa K., Maeta N., Kamiya H. (2018). Understanding
the Colloidal
Stability of Nanoparticle−Ligand Complexes: Design, Synthesis,
and Structure−Function Relationship Studies of Amphiphilic
Small-Molecule Ligands. Chem. Eur J..

[10] Horiguchi G., Uesaka A., Sudo T., Ito Y., Kamiya H., Okada Y. (2023). Flexdispersion: Amphiphilic phosphonic
acid-capped nanoparticles. Colloids Surf., A.

[11] Sudo T., Yamashita S., Koike N., Kamiya H., Okada Y. (2023). Dispersibility
of TiO_2_ Nanoparticles in Less Polar Solvents: Role of Ligand
Tail Structures. Chem. Eur J..

[12] Yamashita S., Ito Y., Kamiya H., Okada Y. (2024). Surface coverage
can control the
dispersibility of TiO_2_ and ZrO_2_ nanoparticles
in hydrophobic solvents: Comparison of linear and branched ligands. Adv. Powder Technol..

[13] Yang Y., Qin H., Jiang M., Lin L., Fu T., Dai X., Zhang Z., Niu Y., Cao H., Jin Y., Zhao F., Peng X. (2016). Entropic Ligands for Nanocrystals:
From Unexpected Solution Properties to Outstanding Processability. Nano Lett..

[14] Monego D., Kister T., Kirkwood N., Mulvaney P., Widmer-Cooper A., Kraus T. (2018). Colloidal Stability of Apolar Nanoparticles:
Role of Ligand Length. Langmuir.

[15] Chew A. K., Van Lehn R. C. (2018). Effect of Core Morphology
on the Structural Asymmetry
of Alkanethiol Monolayer-Protected Gold Nanoparticles. J. Phys. Chem. C.

[16] Li C., Liu L., Zhang Z., Zhang D., Yi S., Yang H., Fan Z. (2023). Anisotropy in Near-Spherical Colloidal
Nanoparticles. ACS Nano.

[17] Monego D., Kister T., Kirkwood N., Doblas D., Mulvaney P., Kraus T., Widmer-Cooper A. (2020). When like
Destabilizes Like: Inverted
Solvent Effects in Apolar Nanoparticle Dispersions. ACS Nano.

[18] Tomai T., Tajima N., Kimura M., Yoko A., Seong G., Adschiri T. (2021). Solvent accommodation effect on dispersibility of metal
oxide nanoparticle with chemisorbed organic shell. J. Colloid Interface Sci..

[19] Lohman B. C., Powell J. A., Cingarapu S., Aakeroy C. B., Chakrabarti A., Klabunde K. J., Law B. M., Sorensen C. M. (2012). Solubility of gold
nanoparticles as a function of ligand shell and alkane solvent. Phys. Chem. Chem. Phys..

[20] Arita T., Yoo J., Adschiri T. (2011). Relation between the Solution-State Behavior of Self-Assembled
Monolayers on Nanoparticles and Dispersion of Nanoparticles in Organic
Solvents. J. Phys. Chem. C.

[21] Usune S., Ando M., Kubo M., Tsukada T., Sugioka K.-I., Koike O., Tatsumi R., Fujita M., Takami S., Adschiri T. (2018). Numerical Simulation of Dispersion and Aggregation
Behavior of Surface-modified Nanoparticles in Organic Solvents. J. Chem. Eng. Jpn..

[22] Saito T., Shoji E., Kubo M., Tsukada T., Kikugawa G., Surblys D. (2021). Evaluation of the work of adhesion at the interface
between a surface-modified metal oxide and an organic solvent using
molecular dynamics simulations. J. Chem. Phys..

[23] Saito T., Takebayashi R., Kubo M., Tsukada T., Shoji E., Kikugawa G., Surblys D. (2022). Effect of surface modifier and solvent
on the affinity between the surface-modified solid and organic solvent:
A molecular dynamics study. AIP Adv..

[24] Saito T., Kubo M., Tsukada T., Shoji E., Kikugawa G., Surblys D., Kubo M. (2023). Molecular dynamics simulations for
interfacial structure and affinity between carboxylic acid-modified
Al_2_O_3_ and polymer melts. J. Chem. Phys..

[25] Sato Y., Kurosawa Y., Saito T., Shoji E., Kikugawa G., Surblys D., Komiya A., Tomai T., Kubo M. (2025). Evaluation
of interfacial affinity between surface-modified metal oxide and solvent
mixture using molecular dynamics simulations. J. Chem. Phys..

[26] Bistafa C., Surblys D., Kusudo H., Yamaguchi Y. (2021). Water on hydroxylated
silica surfaces: work of adhesion, interfacial entropy, and droplet
wetting. J. Chem. Phys..

[27] Yan T., Fichthorn K. A. (2021). Self-Assembly of a Linear Alkylamine Bilayer around
a Cu Nanocrystal: Molecular Dynamics. J. Phys.
Chem. B.

[28] Tobita M., Yasuda Y. (2008). Theoretical and Experimental Vibrational Characterizations
of Amine-Coated Silver Nanoparticles. J. Phys.
Chem. C.

[29] Hughes Z.
E., Wright L. B., Walsh T. R. (2013). Biomolecular Adsorption at Aqueous
Silver Interfaces: First-Principles Calculations, Polarizable Force-Field
Simulations, and Comparisons with Gold. Langmuir.

[30] Tavanti T., Pedone A., Matteini P., Menziani M. C. (2017). Computational Insight
into the Interaction of Cytochrome C with Wet and PVP-Coated Ag Surfaces. J. Phys. Chem. B.

[31] Jorgensen W. L., Maxwell D. S., Tirado-Rives J. (1996). Development
and Testing of the OPLS
All-Atom Force Field on Conformational Energetics and Properties of
Organic Liquids. J. Am. Chem. Soc..

[32] Rizzo R. C., Jorgensen W. L. (1999). OPLS All-Atom
Model for Amines: Resolution of the Amine
Hydration Problem. J. Am. Chem. Soc..

[33] Price M. L. P., Ostrovsky D., Jorgensen W. L. (2001). Gas-phase and liquid-state properties
of esters, nitriles, and nitro compounds with the OPLS-AA force field. J. Comput. Chem..

[34] Siu S. W. I., Pluhackova K., Böckmann R. A. (2012). Optimization of the OPLS-AA Force
Field for Long Hydrocarbons. J. Chem. Theory
Comput..

[35] Terrill R.
H., Tanzer T. A., Bohn P. W. (1998). Structural Evolution of Hexadecanethiol
Monolayers on Gold during Assembly: Substrate and Concentration Dependence
of Monolayer Structure and Crystallinity. Langmuir.

[36] Yen W.-T., Wang K.-H., Yoshida M., Balamurugan M., Kawai T., Venkatesan S., Lee Y.-L. (2021). Self-assembly behavior
and monolayer characteristics of dodecylamine on Au (111) surface. J. Taiwan Inst. Chem. Eng..

[37] Martinez L., Andrade R., Birgin E. G., Martínez J. M. (2009). PACKMOL:
A package for building initial configurations for molecular dynamics
simulations. J. Comput. Chem..

[38] Linstrom, P. J. ; Mallard, W. G. NIST Standard Reference Database Number 69; National Institute of Standards and Technology, 2023.

[39] Shinoda W., Shiga M., Mikami M. (2004). Rapid estimation of
elastic constants
by molecular dynamics simulation under constant stress. Phys. Rev. B.

[40] Tuckerman M., Berne B. J., Martyna G. J. (1992). Reversible
multiple time scale molecular
dynamics. J. Chem. Phys..

[41] Hockney, R. W. ; Eastwood, J. W. Computer Simulation Using Particles, 1 ed.; CRC Press, 2021.

[42] Yeh I.-C., Berkowitz M. L. (1999). Ewald summation for systems with slab geometry. J. Chem. Phys..

[43] Thompson A. P., Aktulga H. M., Berger R., Bolintineanu D. S., Brown W. M., Crozier P. S., in ’t Veld P. J., Kohlmeyer A., Moore S. G., Nguyen T. D., Shan R., Stevens M. J., Tranchida J., Trott C., Plimpton S. J. (2022). LAMMPS
- a flexible simulation tool for particle-based materials modeling
at the atomic, meso, and continuum scales. Comput.
Phys. Commun..

[44] Humphrey W., Dalke A., Schulten K. (1996). VMD: Visual
molecular dynamics. J. Mol. Graphics.

[45] Zhang T., Gans-Forrest A. R., Lee E., Zhang X., Qu C., Pang Y., Sun F., Luo T. (2016). Role of Hydrogen Bonds
in Thermal Transport across Hard/Soft Material Interfaces. ACS Appl. Mater. Interfaces.

[46] Kempfer K., Devémy J., Dequidt A., Couty M., Malfreyt P. (2019). Atomistic
Descriptions of the *cis*-1,4-Polybutadiene/Silica
Interfaces. ACS Appl. Polym. Mater..

[47] Fan H., Wang M., Han D., Zhang J., Zhang J., Wang X. (2020). Enhancement of Interfacial
Thermal Transport between Metal and Organic
Semiconductor Using Self-Assembled Monolayers with Different Terminal
Groups. J. Phys. Chem. C.

[48] Kawagoe Y., Surblys D., Matsubara H., Kikugawa G., Ohara T. (2020). Cross-Plane
and In-Plane Heat Conductions in Layer-by-Layer Membrane: Molecular
Dynamics Study. Langmuir.

[49] Saleman A. R., Chilukoti H. K., Kikugawa G., Shibahara M., Ohara T. (2017). A molecular dynamics
study on the thermal energy transfer and momentum
transfer at the solid-liquid interfaces between gold and sheared liquid
alkanes. Int. J. Therm. Sci..

[50] Benková Z., D S Cordeiro M. N. (2015). Molecular
Dynamics Simulations of Poly­(ethylene oxide)
Grafted onto Silica Immersed in Melt of Homopolymers. Langmuir.

[51] Zhao Y., Qi X., Ma J., Song L., Yang Y., Yang Q. (2018). Interface
of polyimide−silica grafted with different silane coupling
agents: Molecular dynamic simulation. J. Appl.
Polym. Sci..

[52] Leroy F., Müller-Plathe F. (2015). Dry-Surface
Simulation Method for the Determination
of the Work of Adhesion of Solid−Liquid Interfaces. Langmuir.

[53] Kanduč M., Netz R. R. (2017). Atomistic simulations of wetting
properties and water
films on hydrophilic surfaces. J. Chem. Phys..

[54] Uranagase M., Ogata S., Tanaka K., Mori H., Tajima S. (2018). Efficient
scheme for calculating work of adhesion between a liquid and polymer-grafted
substrate. J. Chem. Phys..

[55] Taherian F., Marcon V., van der Vegt N. F. A., Leroy F. (2013). What Is the Contact
Angle of Water on Graphene?. Langmuir.

[56] Pranami G., Lamm M. H. (2015). Estimating Error
in Diffusion Coefficients Derived
from Molecular Dynamics Simulations. J. Chem.
Theory Comput..

[57] De
La Llave E., Clarenc R., Schiffrin D. J., Williams F. J. (2014). Organization of Alkane Amines on a Gold Surface: Structure,
Surface Dipole, and Electron Transfer. J. Phys.
Chem. C.

[58] Mayeda M. K., Kuan W.-F., Young W. S., Lauterbach J. A., Epps T. H. (2012). Controlling Particle
Location with
Mixed Surface Functionalities in Block Copolymer Thin Films. Chem. Mater..

[59] Dahal U., Wang Z., Dormidontova E. E. (2018). Hydration of Spherical PEO-Grafted
Gold Nanoparticles: Curvature and Grafting Density Effect. Macromolecules.

[60] Lin J., Zhang H., Morovati V., Dargazany R. (2017). PEGylation
on mixed monolayer gold nanoparticles: Effect of grafting density,
chain length, and surface curvature. J. Colloid
Interface Sci..

[61] Kister T., Monego D., Mulvaney P., Widmer-Cooper A., Kraus T. (2018). Colloidal Stability of Apolar Nanoparticles:
The Role of Particle
Size and Ligand Shell Structure. ACS Nano.

[62] Widmer-Cooper A., Geissler P. L. (2016). Ligand-Mediated
Interactions between Nanoscale Surfaces
Depend Sensitively and Nonlinearly on Temperature, Facet Dimensions,
and Ligand Coverage. ACS Nano.

