# Supplementary material for: Evaluation of Interfacial Structure and Interaction between Alkylamine-Modified Ag and Organic Solvent via Molecular Dynamics Simulations
Source: Langmuir. 2026 May 12;42(20):14025–33. doi: 10.1021/acs.langmuir.5c06493 (PMC13217611; doi:10.1021/acs.langmuir.5c06493)
Supplement: Supplementary file 1 [file la5c06493_si_001.pdf]

Supporting Information for “Evaluation of Interfacial Structure and Interaction Between Alkylamine-modified Ag and Organic Solvent via Molecular Dynamics Simulations”

Hiroto Sawauchi[a], Yuto Sato[a], Takamasa Saito[a], Eita Shoji[b], Shohei Yamashita[c], Yohei Okada[c], Atsuki Komiya[d], Masaki Kubo[a]*

[a] Department of Chemical Engineering, Graduate School of Engineering, Tohoku University, 6-6-07 Aramaki, Aoba-ku, Sendai, Miyagi, 980-8579, Japan

[b] Department of Mechanical Systems Engineering, Graduate School of Engineering, Tohoku University, 6-6-01 Aramaki, Aoba-ku, Sendai, Miyagi, 980-8579, Japan

[c] Department of Applied Biological Science, Tokyo University of Agriculture and Technology, 3-5-8 Saiwai-cho, Fuchu, Tokyo 183-8509, Japan

[d] Institute of Fluid Science, Tohoku University, 2-1-1 Katahira, Aoba-ku, Sendai, Miyagi, 980-8577, Japan

*Corresponding author: TEL/FAX: +81-22-795-7261; E-mail: m.kubo@tohoku.ac.jp

Piston Wall

To control the pressure perpendicular to the interface, the piston wall was added to a constant external force corresponding to 1 atm in the negative *z*-direction. The position of the piston wall was updated using Newton's classical equation of motion. The interaction between the piston wall and organic solvent was described by the LJ 9-3 potential[1]:

$$U(d) = \varepsilon_{\mathrm{wp}} \left[\frac{2}{15} \left(\frac{\sigma_{\mathrm{wp}}}{d} \right)^9 - \left(\frac{\sigma_{\mathrm{wp}}}{d} \right)^3 \right], \tag{S1}$$

$$\sigma_{\mathrm{wp}} = \frac{\sigma_{\mathrm{Ag}} + \sigma_{\mathrm{C}}}{2}, \tag{S2}$$

$$\varepsilon_{\mathrm{wp}} = \frac{2\pi}{3} \rho_{\mathrm{w}} {\sigma_{\mathrm{wp}}}^3 \sqrt{\varepsilon_{\mathrm{Ag}} \varepsilon_{\mathrm{C}}}, \tag{S3}$$

where ε is the LJ energy parameter, σ is the LJ radius parameter, d is the distance between the piston wall and the carbon atoms, ρ_{w} is the number density of atoms constituting the wall per unit volume, the subscripts Ag and C denote Ag and carbon atoms of organic solvents, respectively. The LJ parameters of Ag were taken from the force field proposed by Heinz *et al.*[2] The wall was assumed to be composed of virtual Ag particles. The mass of the piston wall corresponded to the mass of five layers of Ag (111). To prevent excessive forces on lightweight atoms such as hydrogen, the interaction between the piston wall and the organic solvent was applied only to carbon and oxygen atoms. The cutoff distance for the LJ 9-3 interaction was set to 15 Å, including attractive interactions.

End-to-end Distance of Dodecylamine Ligand

To evaluate the extension and shrinkage of the ligand, the end-to-end distance of the ligand was calculated. The end-to-end distance was defined as the distance between the nitrogen atom of the amine group and the terminal carbon atom of the alkyl chain.

Figure S1 shows the distributions of average end-to-end distance of dodecylamine ligands for different surface coverage. The peak of distance at 25% surface coverage was longer than that at 50% surface coverage because most ligands extended and adsorbed on the Ag surface. For the surface coverages of 50–100%, the higher the surface coverage was, the longer the distance was. No clear difference was observed in the distance distribution between toluene and methanol.

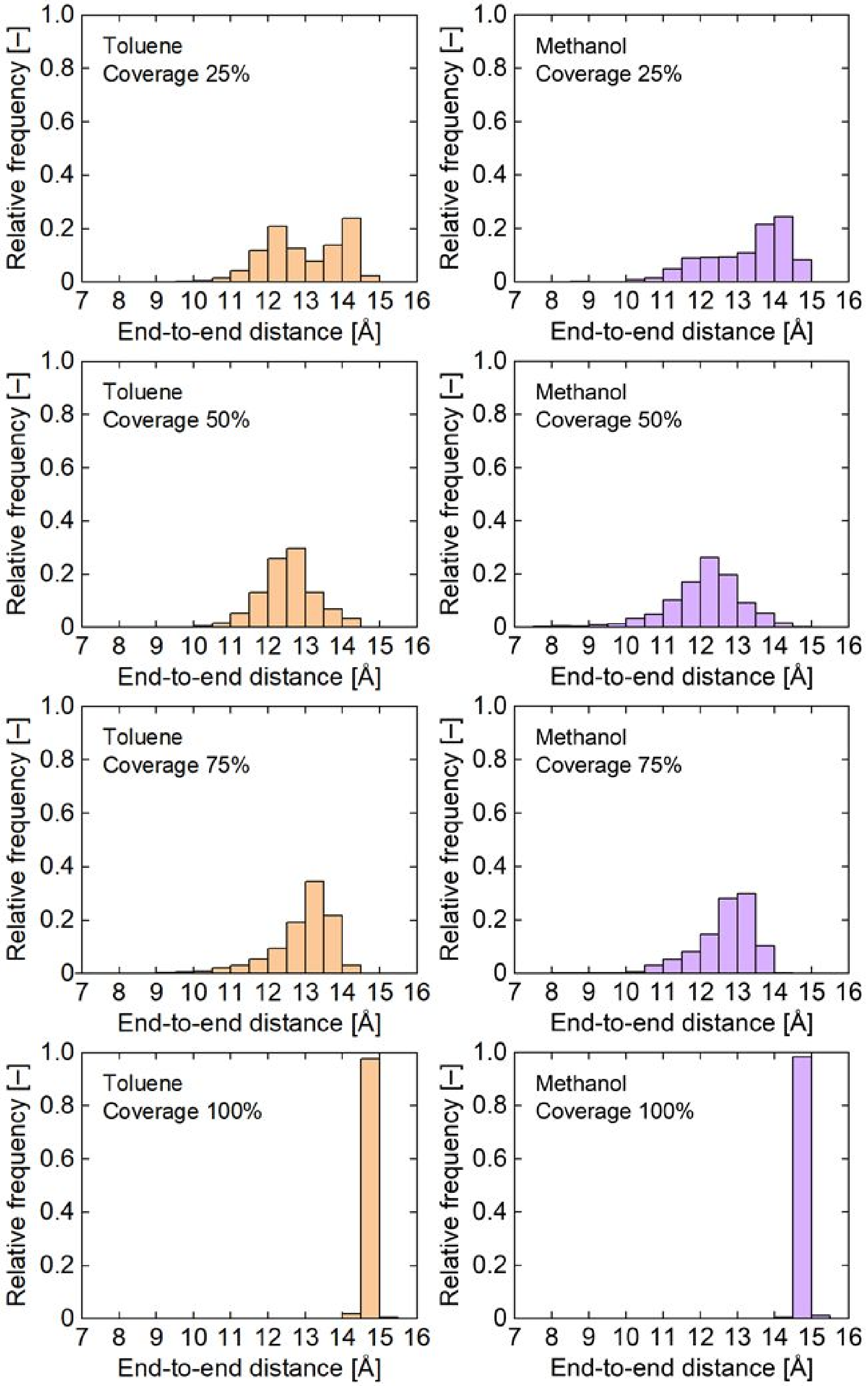

Figure S1. Distributions of average end-to-end distance of dodecylamine ligands for different surface coverage.

Interaction Energy Between Ag and Solvent

The strong adsorption of methanol on the Ag surface is also confirmed by the lower energy of methanol, as shown in Figure S2. For the surface coverage of 0%, the interaction energy between Ag and methanol was lower than that of toluene. For the surface coverage of 25%, the interaction energy between Ag and toluene was almost the same as that of methanol. In the case of toluene, the ligands extended toward the solvent phase, allowing toluene molecules to adsorb onto the exposed Ag surface. Although the interaction between Ag and methanol was stronger, most dodecylamines lay on the Ag surface, limiting the adsorption of methanol on the Ag surface. For the surface coverage of 50–75%, the interaction energy for the methanol system was lower than that for the toluene system. This is because methanol was more adsorbed on the Ag surface than toluene. For the surface coverage of 100%, the interaction energy between Ag and solvents was approximately 0 mJ/m^2.

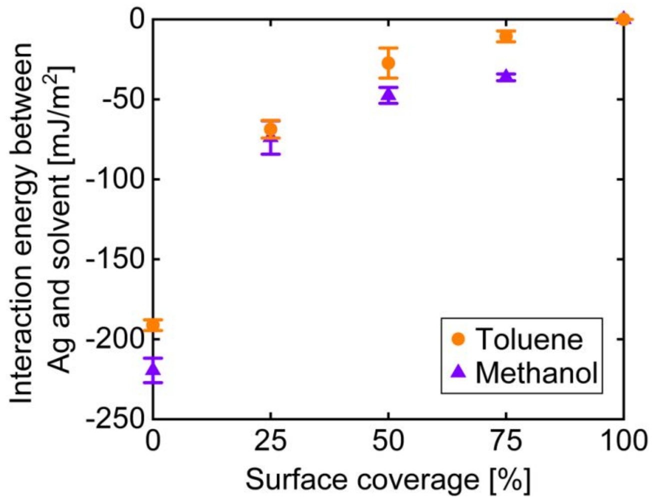

Figure S2. Effect of surface coverage on interaction energy between Ag and solvent for different solvent systems. Error bars represent the standard deviations.

Decomposition of Interaction Energy into LJ and Coulombic Terms

Figure S3 shows the effect of surface coverage on LJ and Coulombic terms of interaction energies for different solvent systems. In the case of toluene, the contribution of LJ terms is dominant, and the contribution of Coulombic terms is not significant for any interaction energies. In the case of methanol, the contribution of Coulombic terms is appeared in the interaction energies with whole ligand and Ag. It indicates that interactions between methanol and amine group or Ag surface were driven by polarity. For the surface coverages of 50–100%, the Coulombic term of interaction energy between alkyl chain of ligand and methanol was positive. It indicates that electrostatic interactions between methanol and nonpolar alkylchain were repulsive.

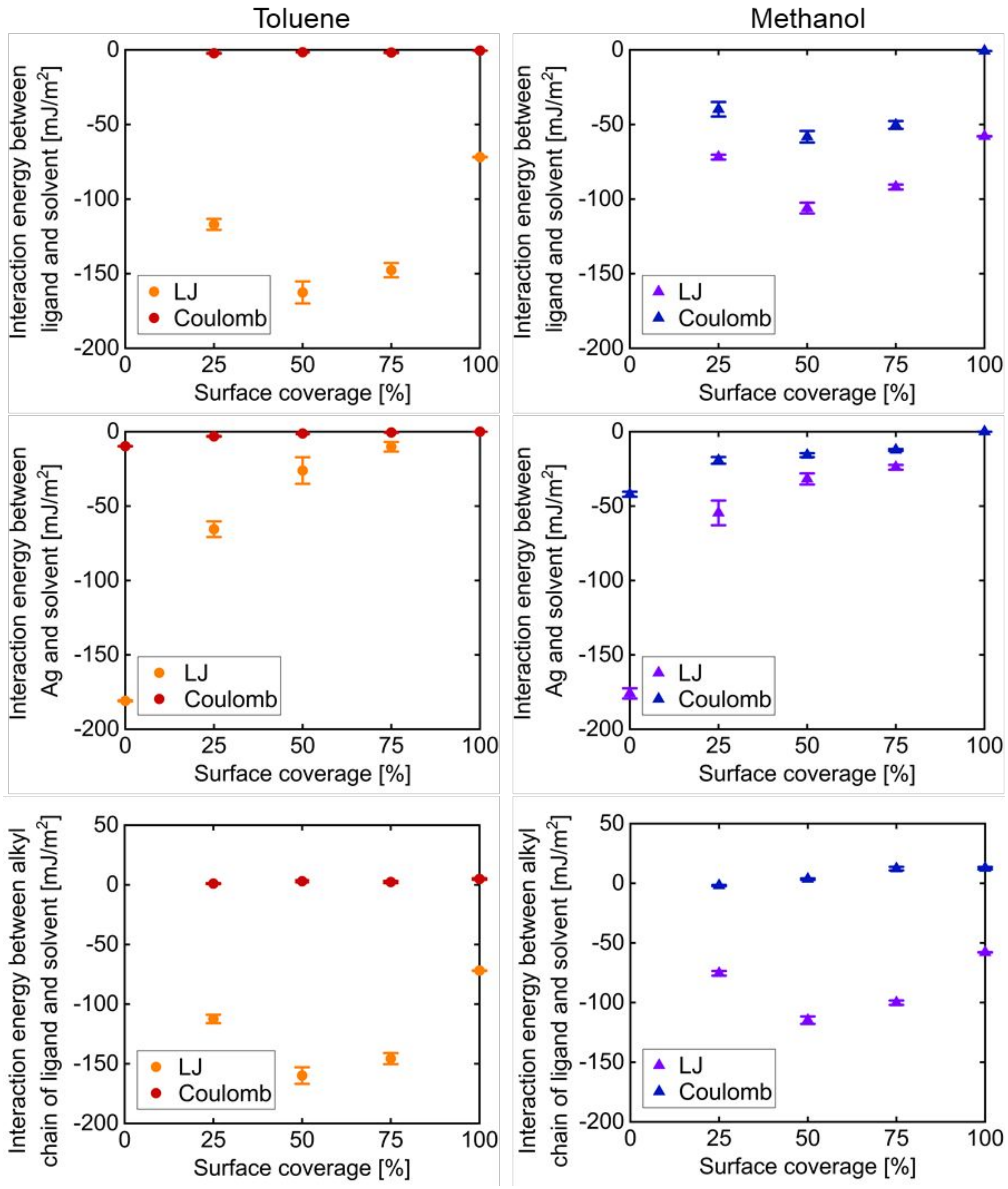

Figure S3. Effect of surface coverage on LJ and Coulombic terms of interaction energies for different solvent systems. Error bars represent the standard deviations.

Statistical Tests

Comparisons between calculated values were evaluated using statistical tests. In this study, uncertainties were estimated using 95% confidence intervals[3]. The following tables show the standard deviations (SD) and 95% confidence intervals (95%CI) of the values in Figures 5–7. The 95% CI was consistently smaller than the SD for all systems, indicating the reliability of the values.

Table S1. Standard deviations (SD) and 95% confidence intervals (95%CI) of the overlap parameter in Figure 5. [$(g/cm^3)^2 \cdot Å$]

	SD				95%CI			
	Coverage				Coverage			
	25%	50%	75%	100%	25%	50%	75%	100%
Toluene	0.1197	0.1597	0.0782	0.0004	0.0663	0.0884	0.0433	0.0002
Methanol	0.2120	0.0742	0.0533	0.0003	0.1174	0.0411	0.0295	0.0001

Table S2. Standard deviations (SD) and 95% confidence intervals (95%CI) of the interaction energy between dodecylamine ligand and solvent in Figure 6. [mJ/m^2]

	SD				95%CI			
	Coverage				Coverage			
	25%	50%	75%	100%	25%	50%	75%	100%
Toluene	3.7504	7.4107	4.8105	0.2423	2.0769	4.1039	2.6639	0.1342
Methanol	5.4716	4.1723	3.5348	0.1548	3.0300	2.3105	1.9575	0.0857

Table S3. Standard deviations (SD) and 95% confidence intervals (95%CI) of the interaction energy between the alkyl chain of the ligand and solvent in Figure 7. [mJ/m^2]

	SD				95%CI			
	Coverage				Coverage			
	25%	50%	75%	100%	25%	50%	75%	100%
Toluene	3.3214	6.3600	4.6233	0.7989	1.8393	3.5221	2.5603	0.4424
Methanol	2.0525	3.4011	2.1976	1.0434	1.1366	1.8835	1.2170	0.5778

The updated Figures 5–7, in which the error bars have been replaced with 95% confidence intervals (95% CI), are shown below. The magnitude of the differences remained comparable between the SD and 95% CI estimates, leading to the same conclusion regarding the similarity of the values.

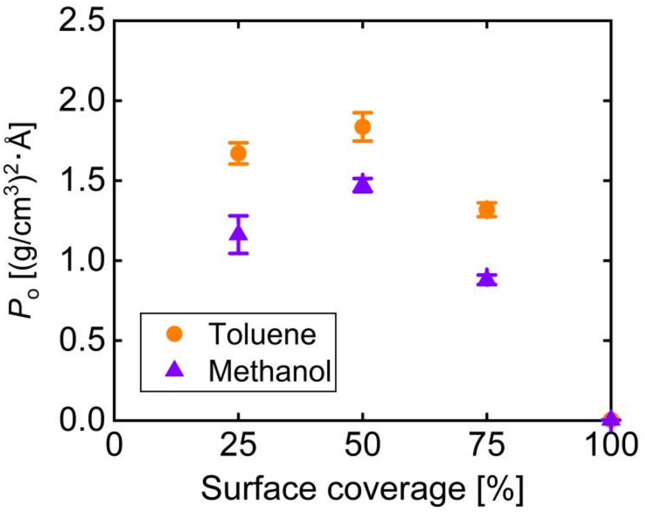

Figure S4. Relationship between the surface coverage and the overlap parameter for different solvent systems. Error bars represent the 95% confidence interval.

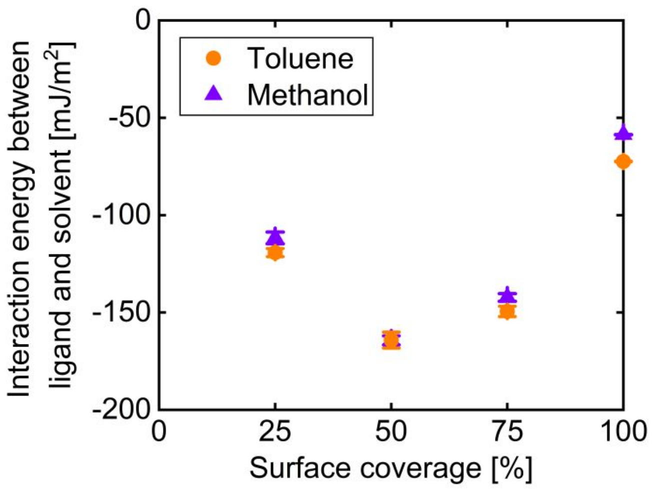

Figure S5. Effect of surface coverage on interaction energy between dodecylamine ligand and solvent. Error bars represent the 95% confidence interval.

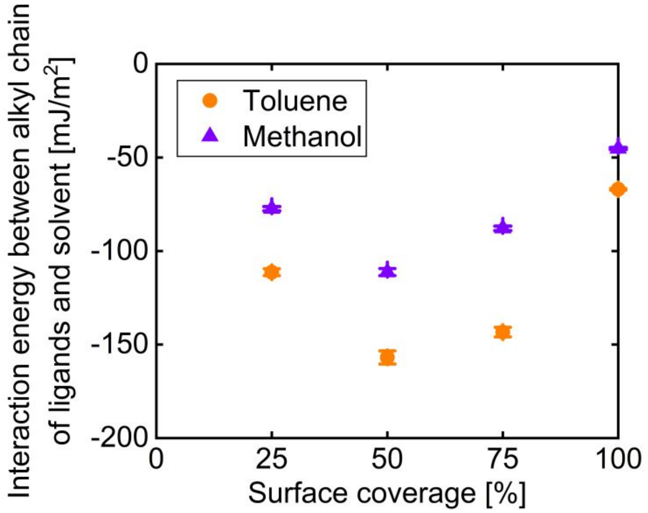

Figure S6. Effect of surface coverage on interaction energy between the alkyl chain of the ligand and the solvent for different solvent systems. Error bars represent the 95% confidence interval.

In addition, Welch's t-test was performed to statistically evaluate the differences of the values shown in Figures 5–7. The following table shows the p-values obtained from Welch's t-test for the differences in values between toluene and methanol in Figures 5–7. In Figures 5 and 7, the values of toluene and methanol exhibited statistically significant differences at each surface coverage ($p < 0.05$). In Figure 6, the significant difference was observed at the other surface coverages but no significant difference was observed at 50% surface coverage.

Table S4. p-values obtained from Welch's t-test for the differences in values between toluene and methanol in Figures 5–7.

	Coverage 25%	Coverage 50%	Coverage 75%	Coverage 100%
Figure 5	8.59×10^{-8}	2.09×10^{-7}	2.49×10^{-15}	7.49×10^{-6}
Figure 6	2.59×10^{-4}	0.957	1.27×10^{-4}	9.21×10^{-39}
Figure 7	6.10×10^{-21}	7.96×10^{-17}	1.08×10^{-20}	5.80×10^{-30}

References

(1) Israelachvili, J. N. *Intermolecular and Surface Forces*, 3rd ed.; Academic Press, 2011.

(2) Heinz, H.; Vaia, R. A.; Farmer, B. L.; Naik, R. R. Accurate Simulation of Surfaces and Interfaces of Face-Centered Cubic Metals Using 12−6 and 9−6 Lennard-Jones Potentials. *The Journal of Physical Chemistry C* **2008**, *112* (44), 17281–17290. DOI: 10.1021/jp801931d

(3) Pranami, G.; Lamm, M. H. Estimating Error in Diffusion Coefficients Derived from Molecular Dynamics Simulations. *Journal of Chemical Theory and Computation* **2015**, *11* (10), 4586–4592. DOI: 10.1021/acs.jctc.5b00574
